# Structural origin of fractional Stokes-Einstein relation in glass-forming liquids

**DOI:** 10.1038/srep39938

**Published:** 2017-01-06

**Authors:** Shaopeng Pan, Z. W. Wu, W. H. Wang, M. Z. Li, Limei Xu

**Affiliations:** 1 International Center for Quantum Materials, School of Physics, Peking University, Beijing 100871, China; 2 Institute of Physics, Chinese Academy of Sciences, Beijing 100190, China; 3 Department of Physics, Beijing Key Laboratory of Opto-electronic Functional Materials & Micro-nano Devices, Renmin University of China, Beijing 100872, China; 4 Collaborative Innovation Center of Quantum Matter, Beijing, China

## Abstract

In many glass-forming liquids, fractional Stokes-Einstein relation (SER) is observed above the glass transition temperature. However, the origin of such phenomenon remains elusive. Using molecular dynamics simulations, we investigate the break- down of SER and the onset of fractional SER in a model of metallic glass-forming liquid. We find that SER breaks down when the size of the largest cluster consisting of trapped atoms starts to increase sharply at which the largest cluster spans half of the simulations box along one direction, and the fractional SER starts to follows when the largest cluster percolates the entire system and forms 3-dimentional network structures. Further analysis based on the percolation theory also confirms that percolation occurs at the onset of the fractional SER. Our results directly link the breakdown of the SER with structure inhomogeneity and onset of the fraction SER with percolation of largest clusters, thus provide a possible picture for the break- down of SER and onset of fractional SER in glass-forming liquids, which is is important for the understanding of the dynamic properties in glass-forming liquids.

The Stokes-Einstein relation (SER) describes the relation between diffusion constant *D* and structural relaxation time *τ*^1^,^2^, which follows *D* ∝ (*τ*/*T*)^−1^ at high temperature *T*. As temperature *T* decreases, the liquids become more and more viscous, thus the increase in relaxation time overtakes the decrease in diffusion, leading to the failure of SER^3^,^4^,^5^,^6^,^7^,^8^. When the SER fails, it has been empirically found that a fractional SER, *D* ∝ (*τ*/*T*)^−*ξ*^ with *ξ* = 0.6 ~ 0.9^9^,^10^,^11^,^12^, holds for a wide range of liquids, such as molecular liquids and atomic liquids^13^,^14^,^15^,^16^,^17^. The breakdown of SER has been considered to be one of the hallmarks of glassy dynamics in liquids. Despite many years of detailed study, the temperature dependence of transport coefficients and structural relaxation times still remains a hot topic of debate^18^,^19^,^20^,^21^. While some studies have shown that the breakdown of SER occurs in a dramatic fashion in the vicinity of the glass transition temperature, there are other studies suggesting its occurrence at a temperature much higher than the mode-coupling temperature^22^,^23^ and at temperatures even higher than the melting temperature^24^. As of yet, there is no agreement on the nature and origin of the breakdown of the SER^4^,^22^,^23^,^24^,^25^.

A commonly proposed explanation for the breakdown of the SER is the presence of dynamic heterogeneity (DH)^26^,^27^, specifically the presence of particles having excessively high and low mobility relative to the average motion, which was observed experimentally^4^,^28^,^29^ and computationally^30^,^31^. There is much current research directed at quantifying the dynamic heterogeneity phenomenon and at understanding its physical origin. However, the association of heterogeneity with the breakdown of SER relation remains no consensus. For instance, Kumar *et al*. pointed that only the mobile particles violate the SER in a hard sphere fluid^32^, while Becker *et al*. found that both the mobile and immobile regions deviate from the SER in a network-forming liquid^14^. More recently, Xu *et al*.^16^ found a correlation between the SER breakdown and the onset of a local structure change in liquids with liquid-liquid phase transformations, however, whether the onset of the fractional SER has a structural origin remains inconclusive.

Using classical molecular dynamics (MD) simulations, we study the breakdown of SER and onset of fractional SER in a model of metallic glass-forming liquid, Cu_64_Zr_36_. We observe the breakdown of SER in the metallic liquid, from normal SER with *ξ* = 1 at high temperatures to fractional SER with *ξ* = 0.67 at low temperatures. This breakdown is correlated with the change in the local structures characterized by the size of the largest cluster consisting of trapped atoms (Fig. 1, see Methods). At high temperatures where SER obeys, the size of the largest cluster is small, indicating a rather homogeneous liquid state. When the size of the largest cluster starts to increase sharply and expand to about half of the simulation box, the SER breaks down. At lower temperatures where the fractional SER follows, the largest cluster percolates the entire system and forms a 3-dimensional network structure. Our results link the SER and fractional SER with structure heterogeneity, thus provide a possible picture for the breakdown of SER and onset of fractional SER in glass-forming liquids.

## Results

### Breakdown of SER and appearance of fractional SER

We first investigate the dynamic properties of the liquid. The temperature dependence of the dynamic behavior between self-diffusion coefficient *D* and *α*-relaxation time *τ* is presented in Fig. 2(a). *D* is computed from the root-mean-square displacement of all atoms^1^ and *α*-relaxation time *τ* is defined as the time when the self-intermediate scattering function decays to 1/*e* for the wave vector *q* = 27.3 *nm*^−1^ (corresponding to the first peak of the static structure factor)^33^. We find that the dynamics of the system follows the SER at high temperatures for *T* > *T*_*SE*_ ~ 1360 K with *ξ* = 1, while it follows a fractional SER at low temperatures for *T* < *T*_*FSE*_ ~ 1160 K with *ξ* = 0.67. We note that the breakdown temperature of SER (1360 *K* < *T* < 1160 *K*) is above the glass transition temperature^34^, thus the mechanism of this SER breakdown is different from the dynamic slowing down near the glass transition. It was previously reported^23^ that the breakdown temperature of the SE relation could be dependent on whether the viscosity (as SE is originally formulated) or relaxation time is chosen. Here, we also present the SER in terms of viscosity (Fig. 2(b)) and find that the SER breakdown temperature using viscosity is the same as using relaxation time.

### SER breakdown and structural heterogeneity

To understand the mechanism of such breakdown in the liquid state, we next study the correlation between local structure and mobility of trapped atoms. For each initial configuration, we classify all the atoms into two groups: trapped or non-trapped. We then calculate the self-intermediate scattering function for each group (trapped or non-trapped). The *α*-relaxation time τ^*^ for each group is calculated as the time at which the corresponding self-intermediate scattering function decays to 1/*e*. We investigated 100 independent initial configurations to obtain the relaxation times for each group. As shown in Fig. 3, the distribution of τ^*^ (statistics from 100 independent realizations), normalized by the relaxation time *τ* of the system at the corresponding temperature, is centered at longer time scale for initially trapped group compared to that for the initially non-trapped group. Thus, the non-trapped atoms which have quasi-nearest neighbors and more free space surrounded move faster compared to the “trapped” atoms which have less free space surrounded (Fig. 3). As temperature decreases, the gap between the relaxation time for the trapped group and non-trapped group becomes larger, leading to a larger difference in the mobility of trapped and non-trapped atoms. This indicates that the local structure inhomogeneity is correlated with local dynamics of the liquid^35^,^36^,^37^,^38^.

To further understand the dynamic breakdown of SER with the structure heterogeneity, we investigate the behavior of clusters consisting of trapped atoms. For each calculation at a fixed temperature, we record the size of the largest cluster consisting of trapped atoms. The temperature dependence of the size of the largest cluster averaged over 100 independent realizations, 〈*S*_*max*_〉, is shown in Fig. 4. At temperatures *T* > 1360 K, *S*_*max*_ is small and more or less constant, indicative of a homogeneous feature of the liquid. The size of the largest cluster, *S*_*max*_, starts to deviate at *T* ~ 1360 K (roughly at the temperature where the SER breaks down), and increase sharply as temperature decreases. This sharp increase is further confirmed from the derivative of *S*_*max*_ with respect to temperature (Fig. 4, inset) with the maximal change in the size of the largest cluster occurring at *T* ~ 1180 K. We note that at the temperature where the *S*_*max*_ has the largest change, the second largest cluster, *S*_2*max*_, is rather small (Fig. 4), indicative of the structure inhomogeneity characterized by the aggregation of small clusters to larger ones. The coincidence of this temperature (~1180 K) with the onset temperature (*T* ~ 1160 K) of the fractional SER is an evidence of the correlation between the structure heterogeneity and the behavior of fractional SER. On the other hand, the size of the *S*_2*max*_ shows a maximum at around 1250 K which is close to the crossover temperature from SER to fractional SER (about 1200 K). At high temperatures, the system has many small but independent clusters. However, as temperature decreases, the largest cluster continues to grow and dominate in space, and other clusters (e.g., the second largest one) become spatially correlated with the largest one. Accordingly, the second largest cluster grows when it is spatially independent of the largest one, but it gradually merges to the largest cluster after the two cluster become spatially correlated. This is why there is a maximum in the size of the second largest cluster at the temperature where the largest cluster starts to dominates, which is related to the crossover temperature from SER to fractional SER. (about 1200 K).

We note that the trapped atom, the fraction of which increases as temperature decrease (e.g., about 11% at 1360 K and 20% at 1160 K, similar to previous studies on the dynamical heterogeneity, typically 5–15%^39^), changes its identity as time involves in the liquid state. Thus, the clusters, consisting of trapped atoms that are dynamically connected in space, is a dynamic feature of the system. Accordingly, the indexes of the atoms inside the clusters also vary with time, which is the consequence of the liquid state. However, in terms of the largest cluster, its size is more or less constant at a fixed temperature. It acts as a characteristic length scale that affects the behavior of the Stokes-Einstein. This is consistent with the picture proposed by Douglas and Hubbard, saying that when the fractional Stokes-Einstein relation obeys, the clusters in supercooled liquids can be the string or sheet-like rather than having a compact structure^40^,^41^. Therefore, the characterization of the structure in terms of the largest clusters consisting of trapped atoms gives a valid statistical picture about the link between structure heterogeneity and Stokes-Einstein relation.

### Fractional SER and percolation of clusters

The next question we ask is what causes the fractional SER at low temperatures. By calculating the normalized projected length of the largest cluster along each dimension, we further investigate the structure heterogeneity characterized by the degree of percolation of the largest cluster consisting of trapped atoms. Let *L*_*pi*_ be the projection of the length of the largest cluster to the *i*th-direction and L_*i*_ be the system size along *i*th-direction. The normalized projected length of the largest cluster in *i*th-direction, *PD*_*i*_, is defined as


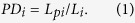


If *PD*_*i*_ = 1, the largest cluster percolates along the *i*th-direction. The temperature dependence of *PD*_*i*_ along the *i*th-direction is shown in Fig. 5. Due to symmetry, *PD*_*i*_ in three directions follows the same temperature dependence. At high temperatures above 1360 K, *PD*_*i*_ increases gradually, indicating that the size of the largest cluster slowly increases as temperature decreases. We note that in the high-temperature region, the system contains many small clusters, but they are not connected as shown in the inset of Fig. 5. At *T* ~ 1360 K, *PD*_*i*_ = 1/2, the largest cluster spans half of the simulation box. As temperature further decreases, the clusters start to get more connected and aggregate to larger ones. At ~1160 K, *PD*_*i*_ ~ 1, the largest cluster percolates along three dimensions thus the entire system, and a network structure consisting of trapped atoms (inset of Fig. 5) is formed. The formation of the network structure occurs at the same temperature where the change in the size of the largest cluster is a maximum (Fig. 4) and the temperature below which the fractional SER obeys. We inspect size effect on *PD* for system size varying from *N* = 200 to 15000. We find that the percolation temperature and SER breakdown temperature for *N* > 5000 are size independent, thus our results for *N* = 10000 are reliable. Therefore, the breakdown of SER has a structure origin. That is, SER follows when the system is homogeneous (consisting of small clusters) at high temperatures, while it breaks down when the structure heterogeneity accumulates in the system characterized by the onset of the sharp increase in the largest cluster. Fractional SER follows when the system forms a network of trapped atoms and the largest cluster percolates the 3-dimensional system, homogeneous in the sense that the majority of atoms are trapped atoms.

Further analysis of the distribution of cluster size, *P(S*), as the function of cluster size, *S*, also confirms the correlation between the percolation of clusters and appearance of the fractional SER. As shown in Fig. 6, the scaled distribution of cluster sizes, P^*^(*S*) = *P(S*)/(*T*/*T*_*c*_ − 1)^*α*^, as function of the scaled cluster size, S^*^ = *S*/(*T*/*T*_*c*_ − 1)^−*β*^, collapse to a master curve, where *α* and *β* are the scaling exponents, and *T*_*c*_ is the critical temperature which also coincides with the onset temperature of fraction SER (~1160 K). As S^*^ → 0, the master curve obeys power-law behavior,


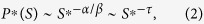


where *α*/*β* is the exponent *τ* in the percolation theory. Since at percolation threshold *T* = *T*_*c*_, S^*^ → 0, according to Eq. 2, this guarantees that the distribution of size of clusters in the entire range of *S* (from 0 to ∞) follows power law behavior,


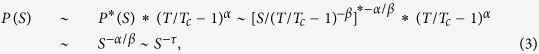


where *τ* is equal to 2.19. According to percolation theory^42^,^43^, at percolation threshold *T*_*c*_, *τ* = *d*/*d*_*f*_ + 1, where *d* is the dimension of the system (=3), and *d*_*f*_ is the dimension of the largest cluster. Using box-counting method, we obtain *d*_*f *_ (~2.40) at *T*_*c*_ ~ 1160 K, which is close to the value of *d*_*f*_ (=2.53) obtained from the scaling theory. This indicates that the distribution of cluster sizes evolves toward a power-law behavior as the system approaches the percolation threshold, thus provides a theoretical evidence of the link between the appearance of fractional SER and the percolation of the trapped atoms in glass-forming liquids.

## Discussion

According to previous studies^14^,^32^, there are two different views regarding the violation of SE relation based on the dynamic heterogeneity due to the existence of subgroup atoms, fast and slow atoms. One is that a “background” of largely immobile regions obeys the normal SE behavior, while mobile regions violate the SE relation and are conjectured to be the cause of SE relation breakdown^32^. The other is that both relatively fast and slow regions violate the SE relation^14^. We investigate the behavior of *D* v.s. *τ*/*T* for trapped and non-trapped atoms, respectively. We find that both trapped and non-trapped atoms follow SE relation at the high-temperature region, but they all violate the SE relation at low temperatures (Fig. 7). Thus, our results are consistent with the latter^14^.

We also test our results in other systems, e.g., Lennard-Jones (LJ) mixture system^33^,^44^ consisting of a binary mixture of classical particles, 8000 type A and 2000 type B with the same mass *m*. Two particles interact with each other via Lennard-Jones potential, *V(r*) = 4*ε*((*σ*/*r*)^12^ − (*σ*/*r*)^6^), where parameters *ε* and *σ* chosen as follows: *ε*_*AA*_ = 1.0, *σ*_*AA*_ = 1.0, *ε*_*AB*_ = 1.5, *σ*_*AB*_ = 0.8, *ε*_*BB*_ = 0.5 and *σ*_*BB*_ = 0.88. In the following, we use *σ*_*AA*_ as the unit of length, 
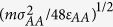
 as the unit of time, and *ε*_*AA*_ as the unit of energy. Constant volume and temperature (NVT-ensemble) is employed in the simulations for the number density 1.2. The dynamic properties (Fig. 8(a)), the temperature dependence of the size of the largest clusters (Fig. 8(b)), and the percolation dimension (Fig. 8(c)) are consistent with our results for metallic systems. That is, the SER breaks down when the size of the largest cluster start to increase sharply and when the largest cluster expands to about half of the system. We note that we are unable to see the onset of the fractional SER as well as the percolation of the size of the largest cluster in this LJ system. This is because in this case the onset temperature of fractional SER at which the largest cluster consisting of trapped atoms percolate is very close to the glass transition temperature^44^ at which we are unable to equilibrium the systems within our simulation time.

Beside the deviation from SER at 1136 K for Cu_64_Zr_36_, we also observe an Arrhenius to non-Arrhenius dynamic crossover at much higher temperature, *T*_*A*_ ≈ 1500 K, as shown in Fig. 9(a). This dynamic crossover is also correlated with structural changes. For instance, the Arrhenius to non-Arrhenius crossover occurs at the temperature at which the number of cluster reaches a maximum shown in Fig. 9(b).

To summarize, using classical MD simulations we study the dynamic and structural properties of a metallic glass-forming liquid, Cu_64_Zr_36_. We find an occurrence of the SER breakdown, from normal SER at high temperatures to a fractional SER at low temperatures. By characterizing the structure of the liquid by the size of clusters consisting of trapped atoms with low mobility, we observe a correlation of the dynamic breakdown of SER with the structural change in the glass-forming liquid. That is, the onset of the breakdown of SER corresponding to the temperature where the size of the largest cluster consisting of trapped atoms starts to increase sharply. In addition, when the largest cluster forms a stable network and percolates the entire system, the dynamics starts to follow a fractional SER. Therefore, our study provides a structural origin of the breakdown of SER in a CuZr metallic glass-forming liquid.

## Methods

### Molecular dynamics

Classical MD simulations are carried out on Cu_64_Zr_36_ metallic liquid using LAMMPS^45^. Our system consists of 6400 Cu atoms and 3600 Zr atoms. The atoms interact via embedded-atom method (EAM) potential function^46^. The system is simulated with periodic boundary conditions. Isothermal-isobaric (NPT)-ensemble simulations using the Nose-Hoover thermostat and barostat are employed in our studies. The equations of motion are integrated with the velocity Verlet algorithm with a time step of 1 fs. Simulations are performed at zero external pressure up to 10 ns along each temperature ranging from 2000 K to 1050 K above the glass transition temperature, more specifically, 1 ns above 1200 K and 10 ns below 1200 K. All our measurement is taken as an average of 100 independent realizations of equilibrated liquid.

### Definitions for trapped atoms and clusters

**Figure 1:**
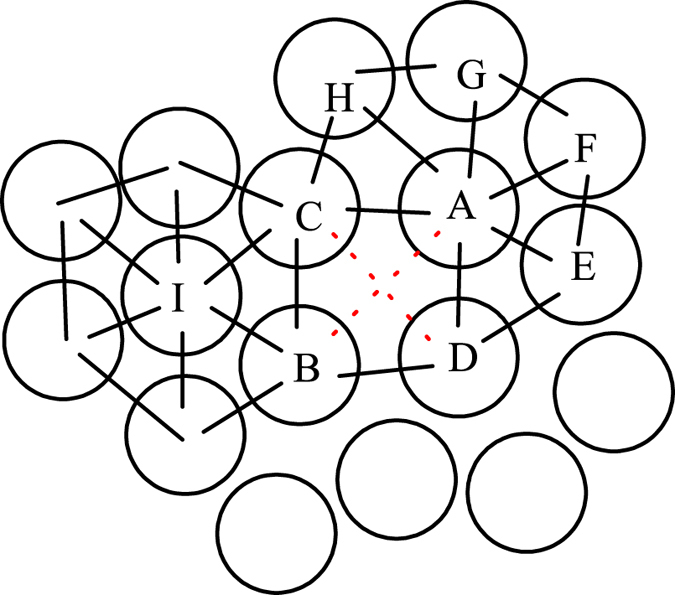
Schematic of “quasi-nearest” and “trapped” atom in a 2-D system. Atoms *C* and *D* belong to “quasi-nearest” atoms since they are an adjacent pair of the nearest neighbors of atom *A* but not nearest neighbors of each other determined by the Voronoi Method. Similarly, atoms *A* and *B* also belong to one pair of “quasi-nearest” atoms. The dark solid line represents the nearest correlation while the red dot line corresponds to the “quasi-nearest” correlation. Atom *I* belongs to “trapped” atom because no pair of quasi-nearest neighbors exists among the nearest neighbors of atom *I*. See Method section.

**Figure 2:**
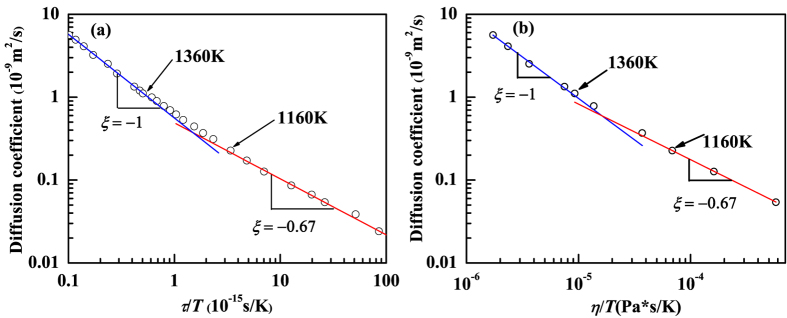
Breakdown of SER and onset of fractional SER. (**a**) Self-diffusion coefficient *D* as function of *α*-relaxation time *τ* and temperature *T. D* follows the SER above 1360 K and obeys fractional SER below 1160 K. (**b**) *D* as function of viscosity *η* and temperature *T*. The breakdown temperature is the same as (**a**).

**Figure 3:**
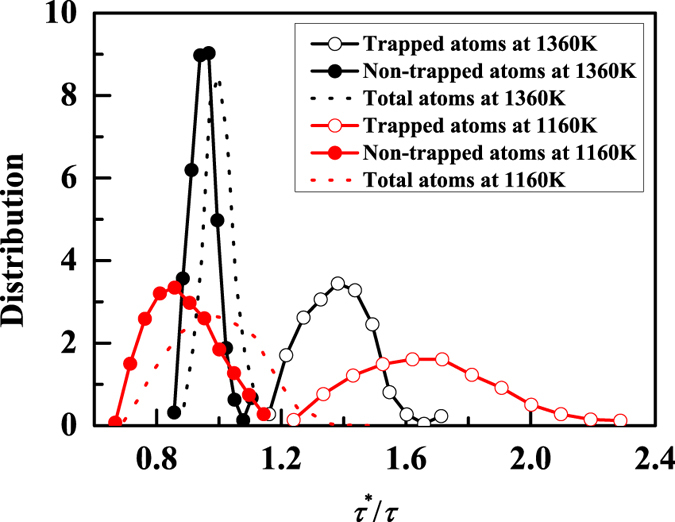
The distributions of the *α*-relaxation time τ^*^ for initially trapped or non-trapped atoms normalized by the *α*-relaxation time *τ* of the system at the corresponding temperatures, respectively. The data for all atoms without classification are also included for comparison.

**Figure 4:**
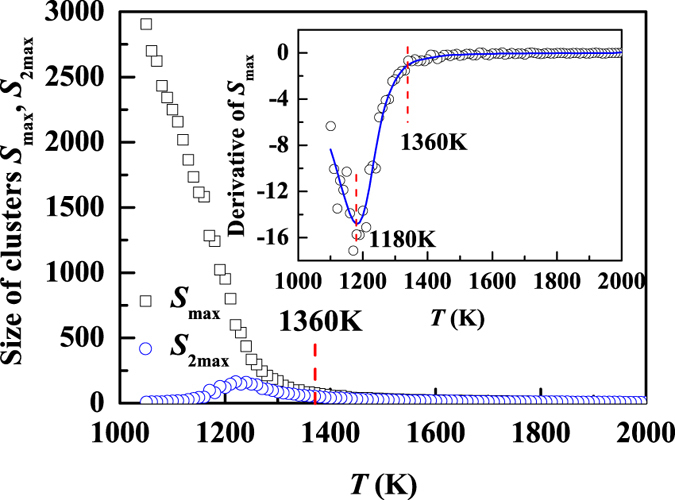
The size of the largest cluster (*S*_*max*_) is more or less a constant at temperatures above 1360 K where the SER follows. The maximal change occurs at 1180 K (inset, derivative of *S*_*max*_ with respect to *T*) near the temperature where the fractional SER starts to follow. To see more clearly the dominance of the largest cluster, the size of the second largest cluster (*S*_2*smax*_) is also presented.

**Figure 5:**
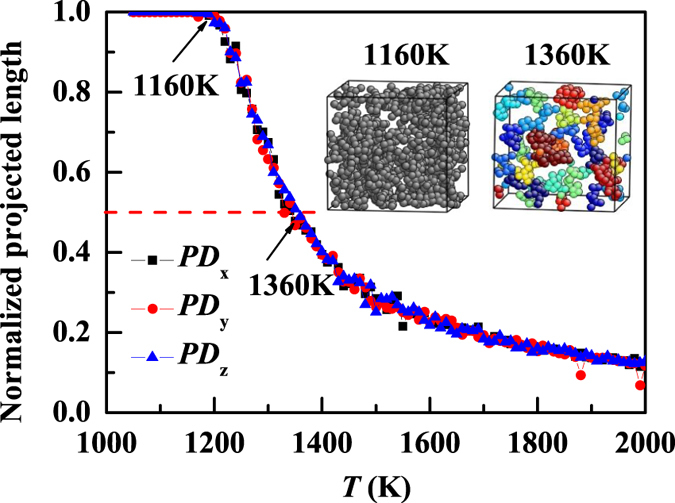
Structural changes in terms of the normalized percolation the length of the largest cluster consisting of trapped atoms along three dimensions. The SER follows when the system is homogeneous dominated by small clusters above 1360 K (inset, right panel), while the fractional SER start to follow below 1160 K when the largest cluster percolate the entire system in 3 dimensions (inset, left panel).

**Figure 6:**
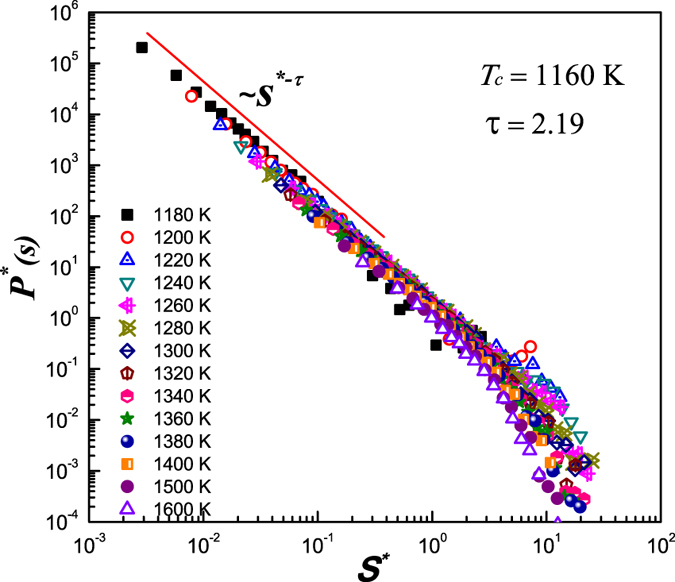
The scaled distribution of the cluster size, P^*^(S^*^) as the function of scaled size of the cluster, S^*^. All the scaled distributions collapsed to a master curve. At percolation threshold, *T*_*c*_ = 1160 K, S^*^ → 0, which guarantees the distribution of cluster size, *P(S*), follows power law behavior for the entire range of *S*, indicative of the correlation between cluster percolation with the onset of fractional SER at *T*_*c*_ ~ 1160 K.

**Figure 7:**
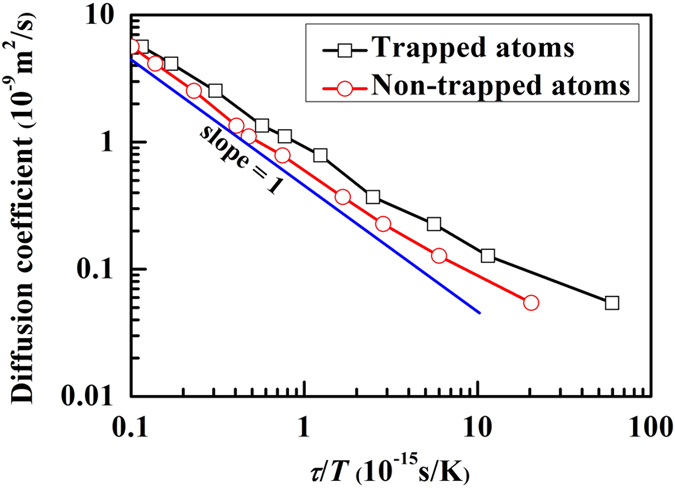
Diffusion coefficient as function of relaxation time and temperature for trapped and non-trapped atoms in CuZr glass-forming liquids. The dynamics of both subgroups follow similar behavior.

**Figure 8:**
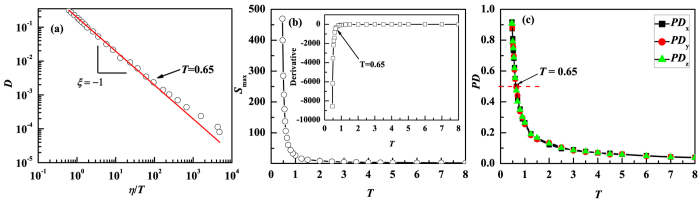
(**a**) Breakdown of SE relation occurs at T = 0.65 in a LJ mixture system. (**b**) The size of the largest cluster as a function of temperature in the LJ mixture systems. SER breaks down when the largest cluster starts to increase sharply at T = 0.65. (**c**) Structural changes in terms of the normalized percolation length of the largest cluster consisting of trapped atoms along three dimensions. At T = 0.65 where SER breaks down, the largest cluster expands to half of the system size.

**Figure 9:**
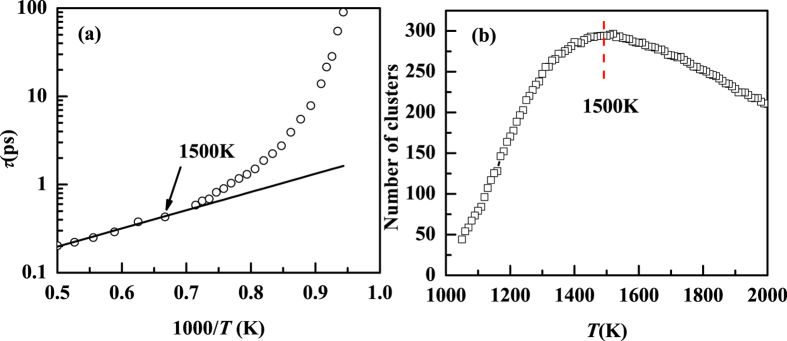
The non-Arrhenius temperature dependence of the relaxation time in Cu_64_Zr_36_. The non-Arrhenius to Arrhenius crossover temperature is higher than the SER breakdown temperature. In our study, the crossover temperature at 1500 K (left panel) coincides with the temperature at which the numbers of clusters is a maximum (right panel).

The local structure of liquid in our study is characterized as follows. For each atom, we first determine its nearest neighbors using Voronoi tessellation method^47^. According to this method, each nearest neighbor of the center atom corresponds to one face of the Voronoi polyhedron. If two Voronoi faces share an edge, the two corresponding atoms among the nearest neighbors of the center atom are defined as an adjacent pair of this center atom^48^,^49^. Next, if the adjacent pair of atoms are not the nearest neighbors of each other, we identify these two atoms as a pair of quasi-nearest atoms. For example, as shown in Fig. 1, atom *C* and atom *D* are a pair of “quasi-nearest” atoms satisfying the following conditions: (i) both are the nearest neighbors of atom *A*; (ii) they are adjacent to each other among the nearest neighbors of atom *A*; and (iii) atoms *C* and *D* are not the nearest neighbors of each other. An atom is called a “trapped” atom (e.g., atom *I* in Fig. 1) if no pair of “quasi-nearest” atoms among its nearest neighbors exist, while the other atoms are non-trapped atoms. A trapped atom has no quasi-nearest atom around while a non-trapped atom has one or more quasi-nearest atoms around. If two “trapped” atoms are the nearest neighbors of each other, they are connected and belong to the same cluster. The size of a cluster is the total number of “trapped” atoms within the cluster. We note that the determination of the nearest neighbors is independent of the choice of different cutoff distances.

## Additional Information

**How to cite this article**: Pan, S. *et al*. Structural origin of fractional Stokes-Einstein relation in glass-forming liquids. *Sci. Rep.* **7**, 39938; doi: 10.1038/srep39938 (2017).

**Publisher's note:** Springer Nature remains neutral with regard to jurisdictional claims in published maps and institutional affiliations.

## References

[b1] Egelstaff P. A. An Introduction to the Liquid State (Clarendon Press, 1992).

[b2] Jonas J. & Akai J. A. Transport processes in compressed liquid methanol. J. Chem. Phys. 66, 4946–4950 (1977).

[b3] Chang I. & Sillescu H. Heterogeneity at the glass transition: Translational and rotational self-diffusion. J. Phys. Chem. B 101, 8794–8801 (1997).

[b4] Ediger M. D. Spatially heterogeneous dynamics in supercooled liquids. Annu. Rev. Phys. Chem. 51, 99–128 (2000). 11031277 10.1146/annurev.physchem.51.1.99

[b5] Swallen S. F., Bonvallet P. A., McMahon R. J. & Ediger M. D. Self-diffusion of tris-Naphthylbenzene near the glass transition temperature. Phys. Rev. Lett. 90, 015901 (2003). 12570626 10.1103/PhysRevLett.90.015901

[b6] Chen B., Sigmund E. E. & Halperin W. P. Stokes-Einstein relation in supercooled aqueous solutions of glycerol. Phys. Rev. Lett. 96, 145502 (2006). 16712090 10.1103/PhysRevLett.96.145502

[b7] Han X. J. & Schober H. R. Transport properties and Stokes-Einstein relation in a computer-simulated glass-forming Cu_33.3_Zr_66.7_ melt. Phys. Rev. B 83, 224201 (2011).

[b8] Kawasaki T. & Onuki A. Slow relaxations and stringlike jump motions in fragile glass-forming liquids: Breakdown of the Stokes-Einstein relation. Phys. Rev. E 87, 012312 (2013). 10.1103/PhysRevE.87.012312 23410336

[b9] Schweizer K. S. & Saltzman E. J. Activated hopping, barrier fluctuations, and heterogeneity in glassy suspensions and liquids. J. Phys. Chem. B 108, 19729–19741 (2004).

[b10] Jung Y., Garrahan J. P. & Chandler D. Excitation lines and the breakdown of Stokes-Einstein relations in supercooled liquids. Phys. Rev. E 69, 061205 (2004). 10.1103/PhysRevE.69.061205 15244552

[b11] Pan A. C., Garrahan J. P. & Chandler D. Heterogeneity and growing length scales in the dynamics of kinetically constrained lattice gases in two dimensions. Phys. Rev. E 72, 041106 (2005). 10.1103/PhysRevE.72.041106 16383361

[b12] Douglas J. F. & Leporini D. Obstruction model of the fractional Stokes-Einstein relation in glass-forming liquids. J. Non-Cryst. Solids 235, 137–141 (1998).

[b13] Voronel A., Veliyulin E., Machavariani V. S., Kislink A. & Quitmann D. Fractional Stokes-Einstein law for Ionic transport in liquids. Phys. Rev. Lett. 80, 2630–2633 (1998).

[b14] Becker S. R., Poole P. H. & Starr F. W. Fractional Stokes-Einstein and Debye-Stokes-Einstein relations in a network-forming Liquid. Phys. Rev. Lett. 97, 055901 (2006). 17026116 10.1103/PhysRevLett.97.055901

[b15] Fernandez-Alonso F. . Observation of fractional Stokes-Einstein behavior in the simplest hydrogen-bonded liquid. Phys. Rev. Lett. 98, 077801 (2007). 17359062 10.1103/PhysRevLett.98.077801

[b16] Xu L. . Appearance of a fractional Stokes-Einstein relation in water and a structural interpretation of its onset. Nat. Phys. 5, 565–569 (2009).

[b17] Kumar P. . Relation between the Widom line and the breakdown of the Stokes–Einstein relation in supercooled water. Proc. Natl. Acad. Sci. 104, 9575–9579 (2007). 10.1073/pnas.0605880103 PMC1559731 16938838

[b18] Ediger M. D. & Harrowell P. Perspective: Supercooled liquids and glasses. J. Chem. Phys. 137, 080901 (2012). 22938210 10.1063/1.4747326

[b19] Kivelson S. A. & Tarjus G. In search of a theory of supercooled liquids. Nat. Mater. 7, 831–833 (2008). 18955990 10.1038/nmat2304

[b20] Han X. J., Li J. G. & Schober H. R. High temperature breakdown of the Stokes-Einstein relation in a computer simulated Cu-Zr melt. J. Chem. Phys. 144, 124505 (2016). 27036459 10.1063/1.4944081

[b21] Soklaski R., Tran V., Nussinov Z., Kelton K. F. & Yang L. A locally preferred structure characterises all dynamical regimes of a supercooled liquid. Philos. Mag. 96, 1212–1227 (2016).

[b22] Sastry S., Debenedetti P. G. & Stillinger F. H. Signatures of distinct dynamical regimes in the energy landscape of a glass-forming liquid. Nature (London) 393, 554–557 (1998).

[b23] Sengupta S., Karmakar S., Dasgupta C. & Sastry S. Breakdown of the Stokes-Einstein relation in two, three, and four dimensions. J. Chem. Phys. 138, 12A548 (2013). 10.1063/1.4792356 23556799

[b24] Brillo J., Pommrich A. I. & Meyer A. Relation between Self-diffusion and viscosity in dense liquids: New experimental results from electrostatic levitation. Phys. Rev. Lett. 107, 165902 (2011). 22107404 10.1103/PhysRevLett.107.165902

[b25] Bartsch A., Ratzke K., Meyer A. & Faupel F. Dynamic arrest in multicomponent glass-forming alloys. Phys. Rev. Lett. 104, 195901 (2010). 20866980 10.1103/PhysRevLett.104.195901

[b26] Stillinger F. H. & Hodgon J. A. Translation-rotation paradox for diffusion in fragile glass-forming liquids. Phys. Rev. E 50, 2064–2068 (1994). 10.1103/physreve.50.2064 9962209

[b27] Tarjus G. & Kivelson D. Breakdown of the Stokes-Einstein relation in supercooled liquids. J. Chem. Phys. 103, 3071–3073 (1995).

[b28] Sillescu H. Heterogeneity at the glass transition: a review. J. Non-Cryst. Solids 243, 81–108 (1999).

[b29] Weeks E. R., Crocker J. C., Levitt A. C., Schofield A. & Weitz D. A. Three-dimensional direct imaging of structural relaxation near the colloidal glass transition. Science 287, 627–631 (2000). 10649991 10.1126/science.287.5453.627

[b30] Hurley M. M. & Harrowell P. Kinetic structure of a two-dimensional liquid. Phys. Rev. E 52, 1694–1698 (1995). 10.1103/physreve.52.1694 9963587

[b31] Kob W., Donati C., Plimpton S. J., Poole P. H. & Glotzer S. C. Dynamical heterogeneities in a supercooled Lennard-Jones liquid. Phys. Rev. Lett. 79, 2827–2830 (1997).

[b32] Kumar S. K., Szamel G. & Douglas J. F. Nature of the breakdown in the Stokes-Einstein relationship in a hard sphere fluid. J. Chem. Phys. 124, 214501 (2006). 16774417 10.1063/1.2192769

[b33] Kob W. & Andersen H. C. Testing mode-coupling theory for a supercooled binary Lennard-Jones mixture. II. Intermediate scattering function and dynamic susceptibility. Phys. Rev. E 52, 4134–4153 (1995). 10.1103/physreve.52.4134 9963886

[b34] Mendelev M. I. . Development of suitable interatomic potentials for simulation of liquid and amorphous Cu-Zr alloys. Philos. Mag. 89, 967–987 (2009).

[b35] Widmer-Cooper A. & Harrowell P. Predicting the long-time dynamic heterogeneity in a supercooled liquid on the basis of short-time heterogeneities. Phys. Rev. Lett. 96, 185701 (2006). 16712373 10.1103/PhysRevLett.96.185701

[b36] Wu Z. W., Li M. Z., Wang W. H. & Liu K. X. Correlation between structural relaxation and connectivity of icosahedral clusters in CuZr metallic glass-forming liquids. Phys. Rev. B 88, 054202 (2013).

[b37] Pan S. P., Feng S. D., Qiao J. W., Wang W. M. & Qin J. Y. Correlation between local structure and dynamic heterogeneity in a metallic glass-forming liquid. J. Alloy. Compd. 664, 65–70 (2016).

[b38] Cheng Y. Q., Sheng H. W. & Ma E. Relationship between structure, dynamics, and mechanical properties in metallic glass-forming alloys. Phys. Rev. B 78, 014207 (2008).

[b39] Glotzer S. C. Spatially heterogeneous dynamics in liquids: insights from simulation. J. Non-Cryst. Solids 274, 342–355 (2000).

[b40] Douglas J. F. A dynamic measure of order in structural glasses. Comp. Mater. Sci. 4, 292–308 (1995).

[b41] Douglas J. F. & Hubbard J. B. Semiempirical theory of relaxation: Concentrated polymer solution dynamics. Macromolecules 24, 3163–3317 (1991).

[b42] Christensen K. & Moloney N. R. Complexity and Criticality (Imperial College Press, 2005).

[b43] Wu Z. W. . Critical scaling of icosahedral medium-range order in CuZr metallic glass-forming liquids. Sci. Rep. 6, 35967 (2016). 27779239 10.1038/srep35967 PMC5078788

[b44] Kob W. & Andersen H. C. Testing mode-coupling theory for a supercooled binary Lennard-Jones mixture I: The van Hove correlation function. Phys. Rev. E 51, 4626–4641 (1995). 10.1103/physreve.51.4626 9963176

[b45] Plimpton S. J. Fast parallel algorithms for short-range molecular dynamics. J. Comput. Phys. 117, 1–19 (1995).

[b46] Mendelev M. I., Sordelet D. J. & Kramer M. J. Using atomistic computer simulations to analyze x-ray diffraction data from metallic glasses. J. Appl. Phys. 102, 043501 (2007).

[b47] Medvedev N. N. The algorithm for three-dimensional voronoi polyhedra. J. Comput. Phys. 67, 223–229 (1986).

[b48] Lerner E., Procaccia I. & Zylberg J. Statistical mechanics and dynamics of a three-dimensional glass-forming system. Phys. Rev. Lett. 102, 125701 (2009). 19392296 10.1103/PhysRevLett.102.125701

[b49] Cheng Y. Q. & Ma E. Atomic-level structure and structure-property relationship in metallic glasses. Prog. Mat. Sci. 56, 379–473 (2011).

